# Current End-of-Life Care Needs and Care Practices in Acute Care Hospitals

**DOI:** 10.1155/2011/869302

**Published:** 2011-12-29

**Authors:** Amy J. Thurston, Donna M. Wilson, Jessica A. Hewitt

**Affiliations:** ^1^BscN Program, Grant MacEwan University, City Centre Campus, Robbins Health Learning Centre, 10700-104 Avenue, Edmonton, AB, Canada T5P 4S2; ^2^Faculty of Nursing, University of Alberta, Edmonton, AB, Canada T6G 1C9

## Abstract

A descriptive-comparative study was undertaken to examine current end-of-life care needs and practices in hospital. A chart review for all 1,018 persons who died from August 1, 2008 through July 31, 2009 in two full-service Canadian hospitals was conducted. Most decedents were elderly (73.8%) and urbanite (79.5%), and cancer was the most common diagnosis (36.2%). Only 13.8% had CPR performed at some point during this hospitalization and 8.8% had CPR immediately preceding death, with 87.5% having a DNR order and 30.8% providing an advance directive. Most (97.3%) had one or more life-sustaining technologies in use at the time of death. These figures indicate, when compared to those in a similar mid-1990s Canadian study, that impending death is more often openly recognized and addressed. Technologies continue to be routinely but controversially used. The increased rate of end-stage CPR from 2.9% to 8.8% could reflect a 1994+ shift of expected deaths out of hospital.

## 1. Introduction

In Canada and many other developed countries, over 50% of deaths each year take place in hospital [1]. Among healthcare professionals, nurses are the most responsible for furthering the possibility of “good” deaths in hospital. Hospital nurses are not only directly accountable for providing hands-on care and for advocating for appropriate end-of-life (EOL) care as staff nurses and as clinical nurse specialists or nurse practitioners, but nurse managers have additional responsibilities to ensure that high-quality EOL care is consistently provided. Many recent health system and other changes could have impacted the care of dying persons in hospital [2–4]. The shift from inpatient-based to ambulatory or outpatient care and the many technological advancements to date are of particular note, as these have contributed to shorter and more acute inpatient episodes, changes that could threaten the opportunity to recognize active dying processes and devise appropriate EOL care plans. Unfortunately, a relatively recent study revealed that dying hospital patients have higher rates of unmet needs as compared to those who die at home [5]. An understanding of current EOL care needs and care practices in hospital is thus needed for hospital quality assurance and improvement purposes. To address this need, a descriptive-comparative study was undertaken in two accessible full-service western-Canadian acute care hospitals. All charts for patients who died from August 1, 2008 through July 31, 2009 were examined using a standardized (post-death) review process. Although the findings of this study cannot be generalized to other hospitals in Canada or elsewhere, this study provides current evidence for broad-based reflection. Reflection on trends is also possible as the findings of this study are compared to a similar mid-1990s Canadian study of EOL care needs and provided care [4].

## 2. Background and Significance of Study

Most deaths in developed countries occur now in advanced old age and typically at the end of a noticeable decline in health with one or more chronic incurable illnesses [6]. It is not surprising then that 80–95% of all deaths in Canada and other developed countries are now considered “expected” or anticipated because of a clearly evident and irreversible declining health state [7]. Only a small proportion of deaths now are due to sudden catastrophic illnesses or accidents, with these unexpected deaths providing much less opportunity to plan or prepare for and potentially enable good deaths.

Good deaths have become a major research focus [8], with a rapidly growing number of studies seeking to determine the factors that contribute to good deaths or that define a good death [9]. To date, many factors have been identified as important or potentially important, including dying at home or in your home community [9]. What needs to be avoided for good deaths to occur is also becoming evident. An enduring concern exists about the overuse of life-prolonging technologies and treatments in hospital [10]. It is imperative that hospital EOL care be as humane as possible, as life-extending care could be in direct opposition to the care needs and care preferences of terminally ill or dying hospital patients [10].

## 3. Methods

Despite some data issues and other limitations, chart reviews have been a common method for many years of acquiring information that is useful for healthcare policy and practice changes [11]. Chart reviews allow for many data variables to be studied over the routinely collected minimum hospital dataset information. Chart reviews are also common in palliative and EOL research because concurrent studies could burden dying persons and their caregivers. As indicated above, a chart review in two full-service hospitals was conducted in one Canadian city. The charts of all persons who died in these two hospitals over a recent 12-month period were reviewed following research ethics approval from a university's health research ethics committee, hospital administrative approval, and the receipt of grant funding to permit data collection. Individual-anonymous data were collected to answer three questions.

1. What are the characteristics of the patients who died in these hospitals?
2. What care was needed and provided before death in hospital and did this care differ in relation to select patient characteristics?
3. What proportion of decedents had an expected death, and was care appropriate for expected versus unexpected deaths?

As outlined in the appended data variables, a wide range of socio-demographic, care needs and care data were of interest. The data variables listed were decided upon by the principal investigator and co-investigator after a review of the literature and after a search of five charts to determine what data existed and were thus possible to collect from the charts. Following this, the data were collected by a research assistant who was directly supervised by the principal investigator. To ensure that correct data were collected, the principal investigator and the research assistant first independently collected data from the same five charts and then compared their findings. Any differences were discussed and rectified, using the charts as reference points. Following this, ongoing monitoring of the data collection process and data were maintained with the data collected also assessed by the co-investigator for completeness and possible data errors. All chart data were entered into a computer spreadsheet and analyzed using SPSS (version 18). Statistical testing primarily involved basic descriptive statistics and bivariate tests, as comparisons were made (chi-square [*χ* ^2^] primarily, as appropriate for the level of variables).

## 4. Results and Discussion

### 4.1. Patient Characteristics

A total of 1,018 persons died in the two hospitals, with all 1,018 charts reviewed. The majority (78.9%) were admitted to hospital via the emergency room (ER) and 92 (9.0%) died in the ER. Hospital stays averaged 19.9 days, with stays ranging from 1 to 367 days (median = 9, SD = 32.0); however, the most common stay was 1 day (11.5%), followed by 2 days (7.8%), 3 days (6.6%), and 4 days (6.0%). Most (88.4%) patients had no surgery performed. In 2/3 cases (68.6%), a family member was present at the time of death. Deaths were unevenly distributed over the year, with more occurring in the winter months and fewer in the summer months, as illustrated by a high of 10.8% in March and low of 6.4% in August. Friday had the highest share of deaths (16.6%) and Sunday the lowest share (12.6%). The greatest proportion of deaths (28.0%) occurred in the hour of 0300–0359. The mean age of decedents was 72.5 (range = birth − 101, median = 77), with the majority aged 65+ (73.8%) and most having been urbanites (79.5%) who resided in the city where the two hospitals were located. A slight majority were male (53.0%) and not married (53.0%). The most common primary diagnosis was cancer (36.2%), followed by cardiovascular disease (23.0%) and respiratory disease (18.8%). Comorbidities were common (mean = 5.9, median = 5, range = 0–25).

### 4.2. EOL Care Needs and Provided Care

Among all 1,018 decedents, 13.8% had CPR performed at some point during this hospitalization, with only 89 patients (8.8%) having CPR performed immediately prior to death being pronounced. In addition, 16.5% received care in a critical care unit (CCU), while 21.7% died on a palliative care unit (PCU) and 25.7% had a palliative care referral. In contrast, 89.0% had oxygen and 89.0% had an intravenous (IV) infusion in use at the time of death; 97.3% had one or more technologies in use at the EOL. Only 7.0% had one or more technologies withdrawn before death, with these withdrawn at various stages throughout the hospital stays.

As shown in Table 1, considerable differences existed in care needs and provided care among the decedents. Of particular note, patients dying of cardiovascular causes, younger patients, males, and rural patients were more likely to have had CPR performed, both at the end of life and at any time during this hospitalization. Similarly, younger persons, males, rural residents, and those dying of cardiovascular disease were more likely to have received CCU care. Younger patients, rural persons, and those dying of cardiovascular diseases were also the most likely to have one or more technologies withdrawn before death whereas patients dying of cancer were the least likely to have had any technologies withdrawn. Patients who died on a PCU and also those who received a palliative care referral were more often younger, married, and a suburbanite. Patients with a respiratory disorder were more likely to have oxygen in use at the EOL and those with a cardiovascular disorder were more likely to have an IV in use at the EOL. In addition, 77.0% had pain recorded as present on the day of death, with younger patients and patients diagnosed with cancer more likely to be in pain on the day of death. Among all patients who indicated or showed that they had pain on the day of death, 98.6% received analgesia. In the few cases where analgesia was not given, sudden cardiovascular deaths were evident. Analgesic use was only recorded when potent analgesics, such as opioids, were given. Younger patients and patients with cancer were more likely to receive analgesia on the last day of life.

As shown in Table 2, care needs and the care provided differed in relation to the ability to mobilize and perform activities of daily living (ADL). Males and patients dying of cardiovascular causes were more likely to be able to walk unassisted and to perform self care. Younger patients were also more likely to be able to perform self care without assistance. In contrast, older patients, female patients, and patients suffering from all disorders other than cardiovascular illnesses were more likely to be bedridden and/or to need complete or partial ADL care.

### 4.3. Expected versus Unexpected Death Findings

Patient deaths were determined to be expected if a DNR (do-not-resuscitate) order was written before death; 87.5% of all 1,018 patients had this order written in their chart. Hospital policy requires DNR orders to be written when indicated by the clinical state of the patient, and after the patient and/or family have been consulted (whenever possible) or whenever they have indicated that they do not want resuscitation to be attempted. DNR orders were written 1 to 367 days in advance of death occurring, with a mean of 11.9 days and median of 7 days. Most DNR orders were written close to death; however, with 11.9% written within 1 day of death, 11.4% within 2 days, 8.7% within 3 days, 7.3% within 4 days, and 5.0% within 5 days.

As shown in Table 3, patients with a DNR order were more likely to receive care in a PCU and/or receive a palliative care referral. Similarly, patients with a DNR order were less likely to die with an IV infusing, but they were also less likely to have any technologies withdrawn. Patients with DNR orders were considerably more likely to have pain and to receive analgesia as compared to patients without a DNR order. Patients with DNR orders were also much more likely to be bedridden or needing assistance to walk, and they also were more likely to need total ADL care or to require some assistance with ADL as compared to the patients who died without a DNR order.

### 4.4. Discussion of Findings

Although chart reviews do not capture all relevant EOL data, this retrospective descriptive-comparative chart review conducted at two full-service hospitals in a Canadian city revealed much about the EOL care needs and care provided to 1,018 patients who died over a one-year period. The following outlines these findings and compares them to other studies, although primarily Wilson's study, as it was a similar study conducted in 1994 in Canada [4]. That study was confined, however, to the use of CPR, and the presence of DNR orders and technologies at the end of life, with minimal data collected on the patient's age and gender, and their ability to walk and care for self near the end of life.

### 4.5. Discussion of General Findings

Many anticipated findings were realized. More deaths occurred in winter than summer and in the middle of the night shift, findings that likely confirm what many practicing hospital nurses have noticed over the years. The decedents were mostly older, another unsurprising finding, as death typically takes place in old age now in developed countries [6]. As also expected, given population mortality trends [6, 12, 13], the most common diagnosis was cancer, followed by cardiovascular and respiratory-related deaths, and with comorbidities common. These findings help to understand the high proportion (1/3) of patients who provided an advance directive, with these directives potentially demonstrating their personal and likely family awareness of impending death and/or their consideration of the inappropriateness of resuscitation efforts. An increasing desire to die a “natural” death has previously been noted among older persons and persons suffering from end-stage illnesses, with this desire now thought to be leading to more deaths taking place outside of hospital [6].

### 4.6. Discussion of DNR and CPR Findings

A very high proportion (87.5%) had a DNR order written in their chart. These findings collectively show hospital nurses and physicians are increasingly engaging in decisions to withhold CPR whenever resuscitation is not indicated, appropriate, or desired by the patient and/or their family. Although some may have concerns that treatment withdrawing or withholding occurs too often now, and that all patients should have resuscitation routinely attempted, this study found that only 7.0% had any technologies stopped over the course of this final hospitalization. Most deaths in these two hospitals arguably were not due to treatment withdrawal but instead to uninterrupted dying processes.

This conclusion is supported by the finding that only 8.8% had CPR performed immediately prior to death and only 13.8% had CPR performed at some point during this hospitalization. The finding that DNR orders were more often written for patients who were bedridden or needing assistance with mobility and for patients who needed full or partial assistance to complete basic ADLs helps to demonstrate that some basic indicators could be signaling impending and irrevocable death, and thus necessitating actions to prepare for this eventuality. There could be many indicators, however, that contribute to DNR orders being written, including the evidence-based awareness that CPR and other resuscitative measures would have a high probability of either failing completely to sustain life [14], or only serving to extend a dying process and thus potentially increasing pain and suffering near death. Further reading of these charts could perhaps also reveal that a serious and noticeable decline in health had occurred, with this decline being a chief factor for DNR orders in hospital and for advance directives to have been developed by patients before this hospitalization. In Canada, where all citizens have universal access to health services, insurance status and personal income have little bearing on hospital admission rates and what care is provided in hospital. The care provided to dying Canadians in hospital is therefore defined by past customs and also by practices arising from an increasing evidence base.

It was not surprising to find that younger patients, those diagnosed with cardiovascular diseases, and rural patients were less likely to have a written DNR order and more likely to have had CPR performed. Younger patients would tend to have more acute and shorter-term illnesses and greater potential for recovery as compared to older patients. Cardiovascular diseases are marked by acute episodes and they are more unpredictable in terms of treatment response, as compared to cancer where death often occurs after all possible treatments have failed. Respiratory-related deaths are also more predictable than cardiovascular deaths, as respiratory failure typically occurs now after all available treatments are no longer effective at maintaining life. Rural people who receive care in city hospitals are also more likely to be suffering from potentially treatable conditions, as this health state would be responsible for their transfer from smaller rural hospitals and for direct ER admissions. As such, CPR was not an uncommon form of care provided in these two hospitals, but it was more often reserved for persons who would likely benefit from it—essentially younger and rural patients, and those diagnosed with cardiovascular illnesses.

### 4.7. Discussion of Palliative Care Findings

Similarly, the patients who died in a PCU or who received a palliative care referral were more often younger. However, these patients were typically diagnosed with cancer, and cancer has long been known to have severe and difficult to manage pain and other symptoms [15]. As such, it is not surprising that younger persons would be the most likely not only to have CPR performed but also to receive specialized palliative care services before death. At the same time, these findings raise concern for older patients dying of cancer and other causes, as they too could have a high burden of pain and other symptoms.

### 4.8. Discussion of Technology Findings

Although specialized palliative care services were oriented to younger persons and those suffering from cancer, basic technologies were a common form of EOL care for all patients everywhere. Most decedents had an IV infusing (89.0%) and/or oxygen in use (89.0%) when dying. Nearly all (97.3%) had one or more technologies in use at the time of death. This EOL practice routine is very similar to Wilson's finding that 94.2% of dying hospital patients had one or more continuous technologies (mainly oxygen and IV fluids) in use at the time of death [4]. Although it is possible that these life-extending technologies were used to deliver analgesics and to otherwise ease the dying process, their use is highly controversial [16, 17].

Oxygen and IV fluids can extend the dying process, and they can also exacerbate or increase EOL suffering [16, 17]. Oxygen, for instance, is uncomfortable when provided at high flow rates and when masks are placed tightly over the nose and mouth. Oxygen masks can prevent or reduce communication with loved ones and nursing staff, and oxygen masks can mar the facial features and thus final memories of loved ones. IVs can contribute to restlessness and wet beds as renal output often continues well into the dying process. Pulmonary edema can occur or be exacerbated by IV therapy, with restlessness, falls, and other issues as a result. IVs also have the problem that they need to be restarted at times, with all starts painful, and with restraints needed at times to ensure that the IV remains in place. Practicing nurses could possibly identify other concerns with the routine use of IVs and oxygen at the EOL, but they may also identify some value in their use.

### 4.9. Discussion of Basic EOL Care Needs Findings

Nursing research into EOL care practices is obviously needed, and particularly as this study found that a large proportion of dying patients were bedridden or needing assistance to mobilize, and a large proportion of dying patients were either needing complete or partial ADL assistance. As such, it is clear that dying patients typically require a great deal of basic nursing care, and this care has not been acknowledged in hospital staffing patterns or staff requirements. Instead, EOL care appears to be routinely technologically based [4], with concerns then that opportunities for a more individualized and humanized process leading to death are lost.

### 4.10. Discussion of Advance Directive and DNR Findings

This concern about technologically based EOL care could be supported by the finding that the CPR rate has apparently increased since 1994 [4], from 2.9% then to 8.8% now at the time of death, and with 13.8% of current patients receiving CPR at some point during their hospital stay. Although this apparent change may suggest that more people are being aggressively treated in hospital to prevent death, it should be remembered that many more people are dying at home or in nursing homes now [6]. The shift of death and dying out of hospital, or at least expected deaths out of hospital, is illustrated by a comparison of the current study's findings with Wilson's study which found that no patients in 1994 provided an advance directive and only 13.1% of patients then verbalized no-CPR preferences [4]. In the current study, 30.8% of the patients who died in hospital provided a written advance directive. These comparative findings illustrate an increasing realization and acceptance among patients and likely families of imminent and irrevocable death.

The current study's finding of a very high rate of DNR orders and the finding that only 16.5% of patients received care in a CCU at some point during their entire hospital stay are also highly important for indicating that hospital nurses and physicians now are increasingly realizing and accepting states of imminent and irrevocable death. Wilson's previous study found that 27.0% of hospital decedents died in a CCU [4], while the current study found that only 16.5% had received CCU care at some point during their hospital stay. Another key current finding is the exceptionally high provision of analgesia (98.7%) on the last day of life. This finding is in contrast to Wilson's previous study which found that analgesia was given infrequently in the last 1–3 days of life [4]. As such, the findings of the current study suggest that some improvements in EOL care have occurred. Although technologies still appear to be normal practice, and CPR is increasingly used in hospital, there are several indications that impending death and active dying are more often recognized in hospital now. In addition, with this recognition, it would appear now that care measures considered appropriate for the EOL are routinely provided.

## 5. Conclusions

This study, although confined to two Canadian hospitals and thus with findings that cannot be generalized across Canada or outside of Canada, helps to focus attention to death and dying in hospital. In Canada and other countries, a considerable portion of deaths each year occur in hospital, with these deaths expected or unexpected. Differences in care needs and in provided care were found when death was expected versus unexpected. The findings of this study also show, as compared to those reported in a similar mid-1990s Canadian study [4], that impending death is more often openly recognized now by hospital nurses and physicians, as well as terminally ill or dying patients and/or their families. Technologies continue to be routinely but controversially used, as they can extend life and it is not certain that these are effective EOL comfort measures. The increased rate of CPR from 2.9% previously to 8.8% could reflect the 1994+ shift of (primarily expected) deaths out of hospital, with hospital-based EOL care being more complex now that there is a need for a rapid determination that death is not only inevitable but also imminent. This chart review also identified some areas for research attention and/or improvements in EOL care. Given the nature of nursing work, nurses are charged with these future developments—to ensure that EOL care is comforting, and that “letting dying patients die” [18] is not only increasingly possible but these patients also are well supported in hospitals and all other places.

## Figures and Tables

**Table 1:** Comparative patient characteristics and EOL care findings.

	*Diagnosis: (i) Ca (ii) Cv (iii) Resp (iv) AO	Age: (i) 0–64 years (ii) 65+ years	Gender: (i) Male (ii) Female	Marital status: (i) Married (ii) Not married	Residence: (i) Urban (ii) Suburban (iii) Rural

Had CPR performed during hospital stay	Ca: 1.6% Cv: 44.0% Resp: 4.7% AO: 9.9% *χ ^2^ = 242.25, df = 3, *P* = .000	Younger: 18.4% Older: 12.1% *χ ^2^ = 6.58, df = 1, *P* = .010	Male: 17.5% Female: 9.7% *χ ^2^ = 13.15, df = 1, *P* = .000	Married: 14.6% Not: 13.0% *χ* ^2^ = 0.59, df = 1, *P* = .444	Urban: 12.4% Suburb: 10.2% Rural: 33.3% *χ ^2^ = 28.85, df = 2, *P* = .000

Had CPR performed immediately prior to death	Ca: 1.6% Cv: 44.0% Resp: 4.7% AO: 9.9% *χ ^2^ = 242.25, df = 3, *P* = .000	Younger: 18.4% Older: 12.1% *χ ^2^ = 6.58, df = 1, *P* = .010	Male: 17.5% Female: 9.7% *χ ^2^ = 13.15, df = 1, *P* = .000	Married: 14.6% Not: 13.0% *χ* ^2^ = 0.59, df = 1, *P* = .444	Urban: 12.4% Suburb: 10.2% Rural: 33.3% *χ ^2^ = 28.85, df = 2, *P* = .000

Had CCU care	Ca: 3.0% Cv: 29.2% Resp: 21.5% AO: 21.4% *χ ^2^ = 83.45, df = 3, *P* = .000	Younger: 20.7% Older: 15.0% *χ ^2^ = 4.52, df = 1, *P* = .034	Male: 19.2% Female: 13.6% *χ ^2^ = 5.83, df = 1, *P* = .016	Married: 15.9% Not: 17.0% *χ* ^2^ = 0.22, df = 1, *P* = .636	Urban: 13.2% Suburb: 20.3% Rural: 43.2% *χ ^2^ = 49.47, df = 2, *P* = .000

Had PCU care	Ca: 53.9% Cv: 2.6% Resp: 2.6% AO: 4.5% *χ ^2^ = 356.13, df = 3, *P* = .000	Younger: 39.0% Older: 15.5% *χ ^2^ = 63.88, df = 1, *P* = .000	Male: 20.5% Female: 22.9% *χ* ^2^ = .832, df = 1, *P* = .362	Married: 27.2% Not: 16.7% *χ ^2^ = 16.35, df = 1, *P* = .000	Urban: 21.1% Suburb: 29.7% Rural: 14.8% *χ ^2^ = 7.27, df = 2, *P* = .026

Had palliative care referral	Ca: 55.8% Cv: 6.0% Resp: 10.6% AO: 9.4% *χ ^2^ = 276.49, df = 3, *P* = .000	Younger: 35.2% Older: 22.3% *χ ^2^ = 17.09, df = 1, *P* = .000	Male: 25.3% Female: 26.2% *χ* ^2^ = 0.11, df = 1, *P* = .742	Married: 30.4% Not: 21.6% *χ ^2^ = 10.34, df = 1, *P* = .001	Urban: 23.9% Suburb: 39.1% Rural: 22.2% *χ ^2^ = 13.78, df = 2, *P* = .001

Oxygen in use at time of death	Ca: 89.2% Cv: 91.0% Resp: 95.8% AO: 80.8% *χ ^2^ = 25.41, df = 3, *P* = .000	Younger: 87.0% Older: 89.7% *χ* ^2^ = 1.64, df = 1, *P* = .200	Male: 89.8% Female: 88.1% *χ* ^2^ = 0.83, df = 1, *P* = .364	Married: 90.0% Not: 88.1% *χ* ^2^ = 0.85, df = 1, *P* = .357	Urban: 88.5% Suburb: 90.6% Rural: 91.4% *χ* ^2^ = 1.01, df = 2, *P* = .604

Intravenous infusion at time of death	Ca: 86.7% Cv: 93.2% Resp: 91.1% AO: 86.6% *χ ^2^ = 8.22, df = 3, *P* = .042	Younger: 90.6% Older: 88.4% *χ* ^2^ = 1.01, df = 1, *P* = .316	Male: 90.2% Female: 87.6% *χ* ^2^ = 1.75, df = 1, *P* = .186	Married: 89.7% Not: 88.3% *χ* ^2^ = 0.50, df = 1, *P* = .478	Urban: 88.3% Suburb: 90.6% Rural: 93.8% *χ* ^2^ = 2.64, df = 2, *P* = .267

Had 1+ technologies withdrawn	Ca: 1.4% Cv: 11.5% Resp: 10.0% AO: 9.0% *χ ^2^ = 29.37, df = 3, *P* = .000	Younger: 10.2% Older: 5.9% *χ* ^2^ = 5.52, df = 1, *P* = .019	Male: 7.5% Female: 6.4% *χ* ^2^ = 0.52, df = 1, *P* = .471	Married: 6.3% Not: 7.6% *χ* ^2^ = .72, df = 1, *P* = .397	Urban: 5.5% Suburban: 7.8% Rural: 20.9% *χ ^2^ = 27.43, df = 2, *P* = .000

Had pain on last day of life	Ca: 94.6% Cv: 59.7% Resp: 71.7% AO: 70.5% *χ ^2^ = 112.220, df = 3, *P* = .000	Younger: 82.7% Older: 75.0% *χ* ^2^ = 6.643, df = 1, *P* = .010	Male: 75.0% Female: 79.2% *χ ^2^ = 2.497, df = 1, *P* = .114	Married: 78.6% Not: 75.6% *χ* ^2^ = 1.339, df = 1, *P* = .247	Urban: 75.2% Suburb: 82.7% Rural: 81.5% *χ* ^2^ = 4.063, df = 2, *P* = .131

Analgesic provided on last day of life	Ca: 94.6% Cv: 59.4% Resp: 71.7% AO: 71.0% *χ ^2^ = 112.939, df = 3, *P* = .000	Younger: 82.8% Older: 75.0% *χ ^2^ = 6.778, df = 1, *P* = .009	Male: 74.8% Female: 79.4% *χ* ^2^ = 3.051, df = 1, *P* = .081	Married: 78.7% Not: 75.6% *χ* ^2^ = 1.381, df = 1, *P* = .240	Urban: 75.2% Suburb: 85.2% Rural: 82.7% *χ* ^2^ = 7.861, df = 2, *P* = .020

*Ca: Cancer; Cv: Cardiovascular; Resp: Respiratory disease; AO: All other.

**Table 2:** Comparative findings in relation to mobility and activities of daily living.

	*Diagnosis: (i) Ca (ii) Cv (iii) Resp (iv) AO	Age: (i) 0–64 years (ii) 65+ years	Gender: (i) Male (ii) Female	Marital status: (i) Married (ii) Not married	Residence: (i) Urban (ii) Suburban (iii) Rural

Mobility					
Unassisted mobility	Ca: 1.6% Cv: 23.10% Resp: 2.1% AO: 1.8%	Younger: 9.0% Older: 5.9%	Male: 9.2% Female: 3.9%	Married: 7.7% Not: 5.7%	Urban: 6.8% Suburb: 4.7% Rural: 8.6%

Assisted mobility	Ca: 10.8% Cv: 16.7% Resp: 15.2% AO: 12.5%	Younger: 12.7% Older: 13.6%	Male: 13.9% Female: 12.8%	Married: 13.6% Not: 13.1%	Urban: 13.0% Suburb: 17.2% Rural: 11.1%

Bedrest	Ca: 87.5% Cv: 60.3% Resp: 82.7% AO: 85.7%	Younger: 78.3% Older: 80.6%	Male: 76.9% Female: 83.3%	Married: 78.7% Not: 81.1%	Urban: 80.2% Suburb: 78.1% Rural: 80.2%

Comparison test result	*χ ^2^ = 141.727, df = 6, *P* = .000	*χ* ^2^ = 3.123, df = 2, *P* = .210	*χ ^2^ = 12.260, df = 2, *P* = .002	*χ ^2^ = 1.747, df = 2, *P* = .418	*χ ^2^ = 3.104, df = 4, *P* = .541

ADL					
Self care	Ca: 0.8% Cv: 18.4% Resp: 1.0% AO: 0.4%	Younger: 8.2% Older: 3.6%	Male: 7.2% Female: 2.3%	Married: 5.5% Not: 4.3%	Urban: 4.8% Suburb: 3.1% Rural: 7.4%

ADL assisted	Ca: 13.0% Cv: 23.9% Resp: 12.0% AO: 11.7%	Younger: 12.4% Older: 16.0%	Male: 15.1% Female: 15.0%	Married: 14.9% Not: 15.2%	Urban: 15.0% Suburb: 16.4% Rural: 13.6%

Complete ADL care	Ca: 86.2% Cv: 57.7% Resp: 86.9% AO: 87.9%	Younger: 79.4% Older: 80.4%	Male: 77.8% Female: 82.7%	Married: 79.7% Not: 80.6%	Urban: 80.2% Suburb: 80.5% Rural: 79.0%

Comparison test result	*χ ^2^ = 151.338, df = 6, *P* = .000	*χ ^2^ = 10.557, df = 2, *P* = .005	*χ ^2^ = 13.381, df = 2, *P* = .001	*χ* ^2^ = .787, df = 2, *P* = .675	*χ ^2^ = 2.18, df = 4, *P* = .703

*Ca: Cancer; Cv: Cardiovascular; Resp: Respiratory disease; AO: All other.

**Table 3:** Care needs and provided care findings in relation to expected versus unexpected deaths.

	DNR order present (Expected death)	No DNR order (Unexpected death)	Test result
CPR performed during hospital stay	5.8%	69.8%	*χ ^2^ = 380.953, df = 1, *P* = .000
CPR performed immediately prior to death	0.8%	64.6%	*χ ^2^ = 566.774, df = 1, *P* = .000
Had CCU care	15.9%	20.6%	*χ* ^2^ = 1.767, df = 1, *P* = .184
Had PCU care	24.5%	1.6%	*χ ^2^ = 34.492, df = 1, *P* = .000
Had palliative care referral	28.7%	4.7%	*χ ^2^ = 33.481, df = 1, *P* = .000
Oxygen in use at time of death	89.7%	84.3%	*χ* ^2^ = 3.338, df = 1, *P* = .068
Intravenous infusion at time of death	88.2%	94.5%	*χ ^2^ = 4.481, df = 1, *P* = .034
Had 1+ technologies withdrawn	6.9%	7.9%	*χ ^2^ = .196, df = 1, *P* = .658
Had pain on last day of life	82.0%	41.7%	*χ ^2^ = 100.84, df = 1, *P* = .000
Analgesic provided on last day of life	82.3%	40.2%	*χ ^2^ = 111.342, df = 1, *P* = .000
Mobility			
(i) Unassisted mobility (ii) Assisted mobility (iii) Bedrest	29.4% 75.7% 94.3%	37.8% 26.0% 36.2%	*χ ^2^ = 262.343, df = 2, *P* = .000
Activities of daily living			
(i) Self care (ii) Assisted care (iii) Total ADL care	28.9% 67.3% 94.8%	27.6% 39.4% 33.1%	*χ ^2^ = 252.967, df = 2, *P* = .000

*Significant findings.

## Appendix

### Data Variables

Chart number (starting with 1)
Hospital identifier
Personal identifier
Day of week of death
Month of death
Year of death
Time of death
Residence (rural, urban, suburban)
Age
Sex
Marital status
Main diagnosis
Admit type (ER or planned admission)
Number of diagnoses
Length of stay
Living will present, yes or no
DNR order present, yes or not
Number of days between DNR order and death
Withdrawal of technology, yes or no
Sudden death, yes or no
Level of consciousness near death (alert, semiconscious, unconscious)
Expected death, yes or no
Mobility on day of death (walking independently, walking with assistance, or not walking)
ADL requirement (independent ADL, assisted ADL, or total ADL assistance)
Pain 3 days prior to death, yes or no
Pain on day of death, yes or no
Surgery performed during hospitalization, yes or no
ICU/CCU care provided during hospitalization, yes or no
Palliative care unit care provided during hospitalization, yes or no
Palliative care referral during hospitalization, yes or no
Technologies in use (total number and types at time of death)
CPR at end of life, yes or no
CPR prior to end of life, yes or no
CPR during entire stay, yes or no
Analgesic on day of death, yes and types or no
Analgesic 3 days prior to death, yes and types or no
Oxygen in use at time of death, yes or no
IV infusing at time of death, yes or no
Family present at death, yes or no.
